# Alterations in mucosa-associated microbiota in the stomach of patients with gastric cancer

**DOI:** 10.1007/s13402-021-00596-y

**Published:** 2021-03-26

**Authors:** Yilin Deng, Xuewei Ding, Qingyuan Song, Gang Zhao, Lei Han, Bowen Ding, Xianhao Wang, Xishan Hao, Hui Li

**Affiliations:** 1 Department of Gastrointestinal Cancer Biology, Tianjin Medical University Cancer Institute & Hospital, Tianjin, 300060 China; 2 National Clinical Research Center for Cancer, Tianjin, 300060 China; 3 Department of Gastric Cancer, Tianjin Medical University Cancer Institute and Hospital, Tianjin, 300060 China; 4 Department of Computer and Information Science, University of Pennsylvania, PA 19104 Philadelphia, USA; 5 Cancer Molecular Diagnostics Core, Tianjin Medical University Cancer Institute & Hospital, Tianjin, 300060 China

**Keywords:** Chronic gastritis gastric cancer microbiota *Helicobacter pylori*

## Abstract

**Purpose:**

The purpose of this study was to characterize alterations in mucosa-associated microbiota in different anatomical locations of the stomach during gastric cancer progression and to identify associations between *Helicobacter* pylori infection and gastric microbial changes in patients with gastric cancer.

**Methods:**

Twenty-five *H. pylori* negative subjects with chronic gastritis and thirty-four subjects with gastric cancer were recruited, including *H. pylori* negative and positive patients with tumors in the antrum and the corpus. Gastric mucosa-associated microbiota were determined by 16S ribosomal RNA gene sequencing using a 454 sequencing platform.

**Results:**

We found that individuals with chronic gastritis from three different anatomical sites exhibited different microbiota compositions, although the microbial alpha diversity, richness and beta diversity were similar. Compared to patients with chronic gastritis, the gastric microbiota compositions were significantly different at the order level in the antrum and the corpus of patients with gastric cancer, which was dependent on the *H. pylori* infection status. Microbial alpha diversity and species richness, however, were similar between chronic gastritis and gastric cancer cases and independent of *H. pylori* status. The microbial community structure in patients with gastric cancer was distinct from that in patients with chronic gastritis. In addition, we found that the presence of *H. pylori* markedly altered the structure in gastric corpus cancer, but only mildly affected the antrum.

**Conclusion:**

Our data revealed distinct niche-specific microbiota alterations during the progression from gastritis to gastric cancer. These alterations may reflect adaptions of the microbiota to the diverse specific environmental habitats in the stomach, and may play an important, as yet undetermined, role in gastric carcinogenesis.

**Supplementary Information:**

The online version contains supplementary material available at 10.1007/s13402-021-00596-y.

## Introduction

The human gastrointestinal tract harbors a broad range of microbiota which contribute to the establishment of a symbiotic relationship with the host [1]. This relationship provides a nutrient-rich environment for the microbiota, while playing important roles in maintaining physiological balances and health of the host [2–4]. The microbiota in the stomach form a complex community which can be shaped by various factors. Due to the harsh environment in the stomach, it was commonly assumed to be sterile until the discovery and isolation of *Helicobacter pylori* [5]. Recently, the profiles of gastric microbiota have gradually been unveiled by virtue of next generation sequencing (NGS) technologies. *Proteobacteria*, *Firmicutes*, *Bacteroidetes*, *Actinobacteria* and *Fusobacteria* are the most commonly detected phyla in the stomach [6]. Alterations in richness or diversity of microbiota may result in dysbiosis. Increasing evidence indicates that dysbiosis of the gastrointestinal microbiota is associated with gastric cancer [7] as well as with a wide variety of other disorders, including inflammatory bowel disease [8], liver pathology [9], autoimmune disease [10] and brain dysfunction [11].

Gastric cancer is one of the leading causes of cancer-related death worldwide, especially in eastern Asia [12]. A complex interaction between the host genetic background and environmental factors contributes to the development of gastric cancer [13]. Gastric adenocarcinoma comprises more than 90% of all malignant tumors in the stomach, including cardia and non-cardia gastric cancer according to the site where the tumor arises. Risk factors associated with non-cardia gastric cancer are *H. pylori* infection, low socio-economic conditions, and dietary habits such as low intake of vegetables and fruits and high consumption of salty and smoked food. Obesity and gastroesophageal reflux disease are exclusive factors for cardia gastric cancer [14].

*H. pylori* has been designated as a class I carcinogen and has been identified as the main risk factor for gastric carcinoma. Persistent chronic inflammation induced by *H. pylori* infection is a decisive factor in the progression of gastric cancer [13]. However, only a small portion (< 3%) of *H. pylori* infected patients eventually develop gastric cancer [13]. It has been reported that complex gastric microbiota may significantly accelerate the onset of precancerous gastric lesions rather than *H. pylori* infection alone in a transgenic model of gastric carcinogenesis, i.e., insulin-gastrin (INS-GAS) mice [15]. Antimicrobial therapy significantly delayed the onset of gastrointestinal intraepithelial neoplasia (GIN) in both *H. pylori*-infected and *H. pylori*-free INS-GAS mice [15]. In addition, it was found that gastric colonization with a restricted microbiota promoted neoplastic lesions in *H. pylori*-mono-associated INS-GAS mice [16]. So, several lines of evidence indicate a relationship between gastric dysbiosis and *H. pylori* infection, and the development of gastric cancer.

However, due to the different techniques used, the complexity of bioinformatic analyses and the intricacy of gastric cancer, there is currently no consensus on the drivers or modifiers in the microbiome that mediate the development of gastric cancer. It is well known that long-term inflammation in the stomach may lead to chronic gastritis, atrophy, intestinal metaplasia, dysplasia and, ultimately, cancer [17]. The purpose of this study was to characterize alterations in mucosa-associated microbiota during gastric cancer development and to delineate the influence of *H. pylori* infection on the gastric microbial community in gastric cancer patients. Information on the microbial profile in the stomach was obtained by analyzing microbiota in different anatomic sites (antrum, corpus and cardia). Our findings underscore dysbiosis as a risk factor or consequence of gastric cancer and a concomitant influence of *H. pylori* infection on gastric cancer development.

## Materials and methods

### Patients and sample collection

This study was approved by the Clinical Research Ethics Committees of Tianjin Medical University Cancer Institute and Hospital, Tianjin, China. Written informed consent was obtained from all individuals included in this study. All subjects enrolled in this cohort resided in Tianjin, China. Patients were recruited at Tianjin Medical University Cancer Institute and Hospital. *H. pylori* infection status was examined by urease test. Twenty-five individuals (12 female and 13 male, age range: 45 to 70) with chronic superficial gastritis were recruited in this study. All individuals in this group were *H. pylori* negative. Gastric mucosal biopsy samples were obtained from lesions including the antrum (*n* = 10), corpus (*n* = 7) and cardia (*n* = 8) during standard upper gastroenterology endoscopic examination. In addition, thirty-four patients (10 female and 24 male, age range: 46 to 75) with gastric cancer were enrolled in this study. All of them were diagnosed as gastric adenocarcinoma, including patients with cancer in the antrum (*n* = 19) and corpus (*n* = 15). Cancer stages included stage I (*n* = 3), stage II (*n* = 7), stage III (*n* = 23) and stage IV (*n* = 1). Gastric cancer biopsy tissues were obtained from the sites of cancer lesion during surgery. Five patients with gastric antrum cancer and four patients with gastric corpus cancer were *H. pylori* positive. Patients who received antibiotics, acid blockers (including proton pump inhibitors and histamine-2 receptor antagonists), anti-inflammatory drugs or probiotic treatment during the past 6 months were excluded. In addition, none of the patients received chemotherapy or radiotherapy prior to the surgery.

### 16S rRNA gene sequencing and microbiota profile analysis

Frozen gastric mucosa biopsy samples and gastric cancer tissues were sent to the Research and Testing Laboratory, LLC (Lubbock, TX, USA) for metagenomic analyses. Total genomic DNA was extracted and sequenced using primers targeting the 16S ribosomal regions on a Roche 454 sequencing platform. Sequence data were processed using a Research and Testing pipeline that is described at http://www.researchandtesting.com/docs/Data_Analysis_Methodology.pdf. Raw DNA sequencing data were analyzed using QIIME (http://www.qiime.org). The data analysis pipeline consisted of two major stages, a denoising and chimera detection stage and a microbial diversity analysis stage. The process of denoising was used to obtain clean reads by discarding adaptors, short and low-quality sequences. Chimera detection and removal by executing UCHIME on the filtered data served as a denoising method. Next, sequences were clustered into OTUs (Operational Taxonomic Units) using the UPARSE algorithm with at least 97% similarity. Taxonomic information was assigned to centroid sequences from each cluster by the RDP (ribosome database project) classifier against the NCBI database, after which diversity parameters were examined from two perspectives. Alpha diversity analysis was used to describe the richness and diversity of the microbiota within the sample. First, overall richness was quantified using the Chao 1 richness estimator. Second, overall diversity was expressed as Shannon Diversity, which was determined by both richness and evenness, the distribution of abundance among distinct taxa. Beta diversity referred to the diversity among samples, which was measured by covariance matrix and visualized via principal component analysis (PCA). Specific microbial taxa were identified through a linear discriminant analysis effect size (LEfSe) algorithm, performed on the Galaxy web-based interface (http://huttenhower.sph.harvard.edu/galaxy). Taxa with a linear discriminant analysis (LDA) > 3.0 and an alpha value < 0.05 were considered significant.

### Statistical analysis

Data that were not normally distributed are presented as median values and ranges (min to max). Mann-Whitney U test was used to determine the difference between two sample groups. Multiple sample groups were analyzed using Kruskal-Wallis test. Data that were normally distributed are presented as mean ± SEM, and one-way ANOVA was used to analyze differences. All tests were performed using GraphPad Prism 8.0.1. *P < 0.05* was considered statistically significant. Heatmaps and PCAs were generated using Python.

## Results

### Gastric mucosa-associated microbiomes differ in different anatomical locations of individuals with chronic gastritis

**Fig. 1:**
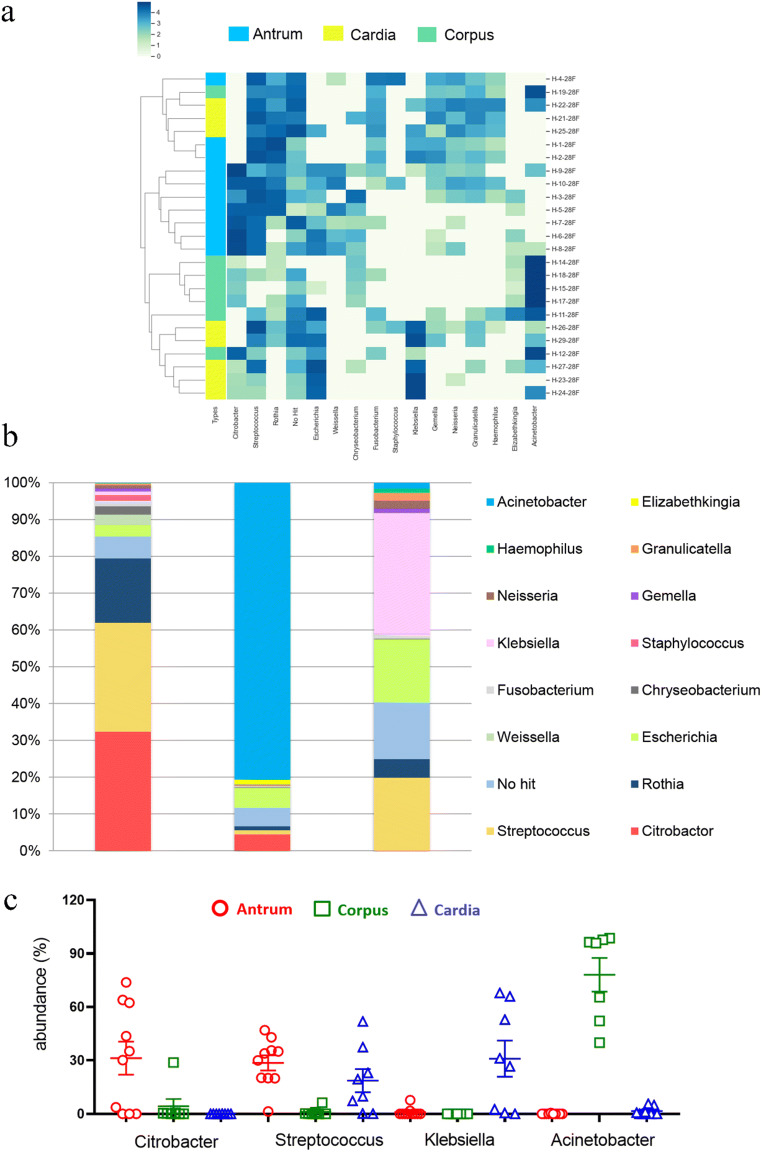
Microbial composition analysis of chronic gastritis patients. **a** Heatmap of gastric mucosa-associated microbiota with relative abundance over 1% in all subjects. **b** Bar plots of relative abundances of gastric mucosa-associated microbiota. **c** Representative microbiota among chronic gastritis subjects from three different anatomical locations

Although microbial profiles in the stomach have been reported before, knowledge on whether different anatomical sites harbor different microbiota, especially under inflammatory conditions, is limited. Therefore, we first analyzed gastric mucosa-associated microbiota in different anatomical sites including the antrum, corpus and cardia in patients with chronic gastritis. The most representative phyla were *Proteobacteria*, *Firmicutes*, *Bacteroidetes*, *Actinobacteria* and *Fusobacteria*, representing over 95% of the total phylum (Table 1). The relative abundances of *Proteobacteria* and *Firmicutes* were significantly different among the three different anatomical sites (*p* < 0.05, Table 1). *Proteobacteria* were predominant in the corpus, whereas those of *Proteobacteria* and *Firmicutes* were at comparable levels in the antrum and cardia (Table 1). At the genus level, *Citrobacter* (31.30 ± 2.65%) was the most abundant taxa in the antrum, followed by *Streptococcus* (28.64 ± 1.22%) and *Rothia* (16.95 ± 1.82%). However, in the corpus, the major genus was *Acinetobacter* (78.06 ± 3.30%), with a relative abundance > 90% in over half of all samples. Conversely, the overrepresented genus in the cardia was *Klebsiella* (30.99 ± 3.36%), followed by *Streptococcus* (18.68 ± 2.15%) and *Escherichia* (16.19 ± 2.18%) (Fig. 1). Consistent with the findings at the genus level, *Acinetobacter sp* and *Klebsiella sp* were the only significantly abundant species enriched in the corpus and the cardia, respectively (Supplementary Fig. 1). *Weissella confuse*, a member of the genus *Weissella*, was detected as a high-abundance species in antrum-predominant gastritis (Supplementary Fig. 1). However, a large degree of inter-subject variability was observed, with relative abundances ranging from 0 to 98.89%.

**Table 1:** Relative abundances of major bacterial phyla in chronic gastritis subjects

Taxonomy	Antrum (%)	Corpus (%)	Cardia (%)	*P* value
*Proteobacteria*	38.03	80.18	48.99	**0.04**
*Firmicutes*	36.94	1.69	28.71	**0.01**
*Actinobacteria*	14.63	1.36	5.6	0.11
*Bacteroidetes*	2.95	7.26	2.13	0.58
*Fusobacteria*	1.7	0.38	1.26	0.91

The *P* values are calculated using Kruskal-Wallis test and marked in bold if < 0.05

The relative abundance of *H. pylori* in all subjects included in this group was extremely low*.* According to a previous report, *H. pylori* sequencing positive was referred to as those samples with a relative abundance >1% [18]. In this group, the *H. pylori* sequence status of all samples was negative (< 1%), consistent with the results of the conventional urease test.

**Fig. 2:**
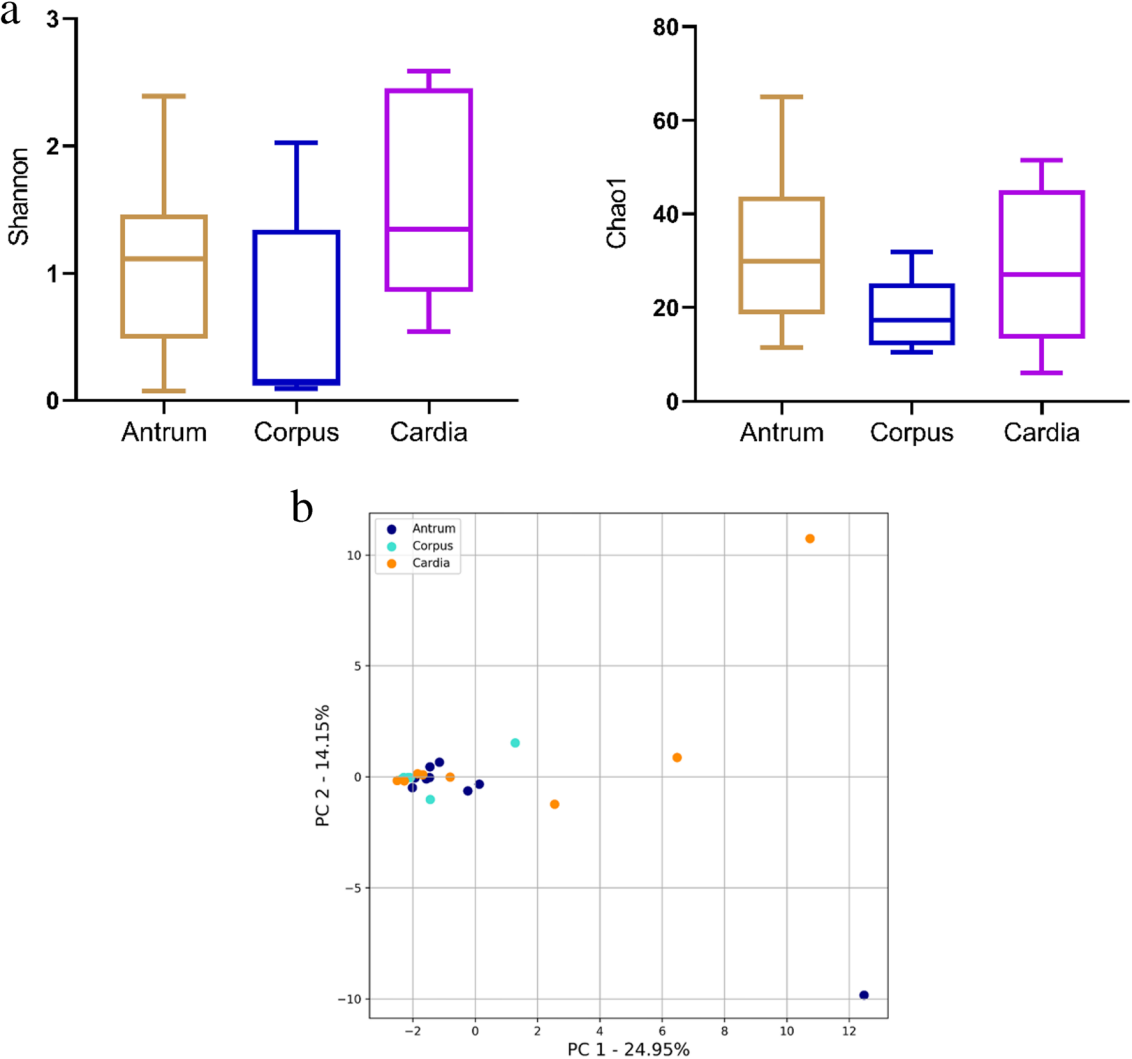
Microbial community structure analysis of chronic gastritis patients from three different anatomical positions. **a** Microbial alpha diversity and richness analysis (*p* > 0.05). **b** Principal component analysis (PCA)

To evaluate differences in microbiota structure of chronic gastritis from three different anatomical sites, we measured microbial alpha and beta diversities. Chao 1 and Shannon indexes were used to measure species richness and alpha diversity, respectively. We found that there was no significant difference in microbial richness and alpha diversity within the three different anatomical sites (Fig. 2a). Differences in microbial community structures of chronic gastritis from three different anatomical sites were assessed by beta diversity and visualized by PCA plot. A PCA plot based on covariance matrix revealed that most samples from the three different anatomical locations overlapped, although the cardia cases were more dispersed than the those of other two sites (Fig. 2b). The three different anatomical sites were not clustered separately, suggesting similarity in the overall community structure of chronic gastritis in the three different anatomical locations.

**Fig. 3:**
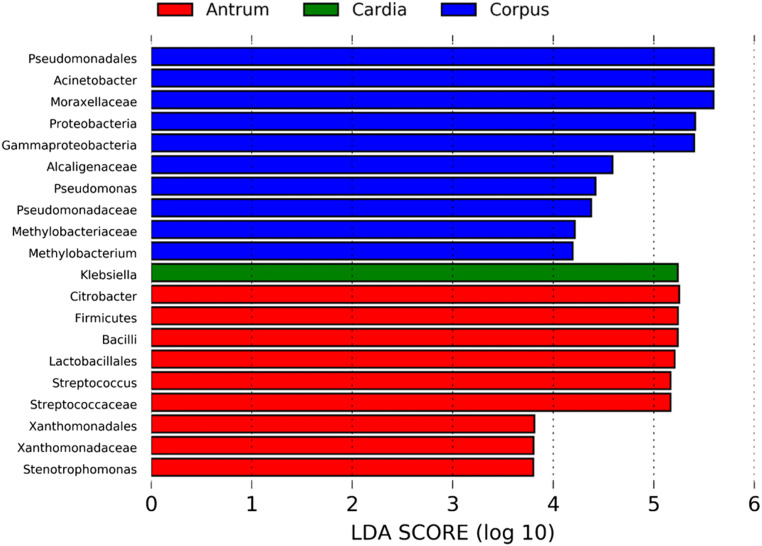
Differential bacteria among three different anatomical sites by LEfSe analysis (LDA scores > 3 and *p* < 0.05)

To further assess specific taxa associated with each anatomical site, we conducted LEfSe analysis. Overall, there were 20 taxa that were differentially abundant in gastritis from the three different anatomical sites. Nine of them were enriched in antrum gastritis, including the phylum *Firmicutes* with its specific genus *Streptococcus*, and the phylum *Proteobacteria* with its specific genera *Stenotrophomonas* and *Citrobacter*. The genus *Klebsiella*, within the phylum *Proteobacteria*, was the only enriched taxa in the cardia. All taxa enriched in the corpus belonged to the phylum *Proteobacteria*, including the genera *Pseudomonas*, *Acinetobacter* and *Methylobacterium* (Fig. 3). These results indicate that the most relevant taxa are responsible for the differences in chronic gastritis from the three different anatomical sites, and that all of them belong to the phyla *Firmicutes* and *Proteobacteria*.

Taken together, these data suggest that the composition of the microbiota in the different anatomical sites varies, but that there are no differences in microbial diversity and evenness in individuals with chronic gastritis.

### Differences in the gastric mucosa-associated microbiome in *H. pylori* negative patients with gastric cancer

**Fig. 4:**
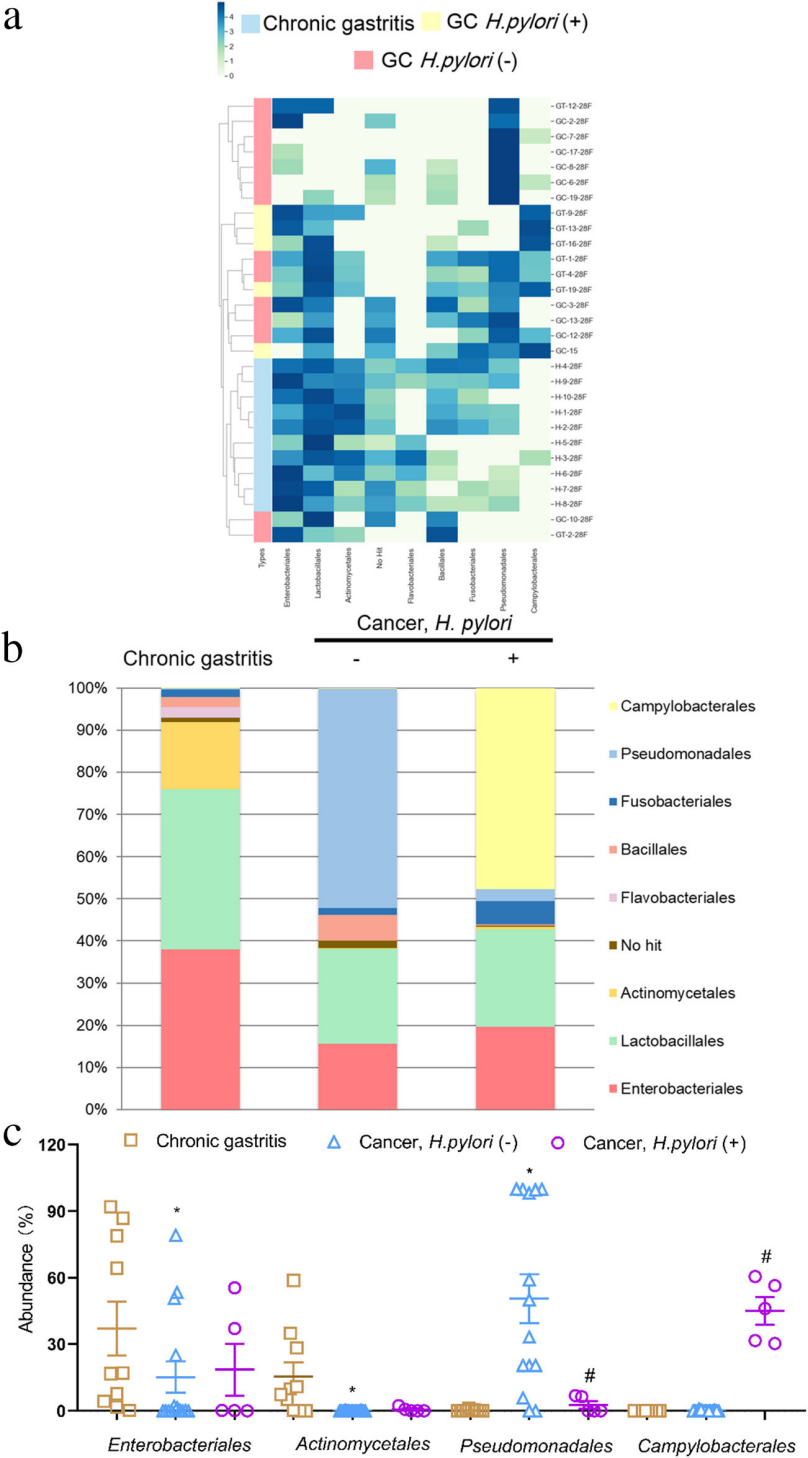
Microbial compositional analysis of patients with antrum predominant gastritis and gastric antrum cancer. **a** Heatmap of gastric mucosa-associated microbiota with relative abundance over 1% in all subjects. **b** Bar plots of relative abundance of gastric mucosa-associated microbiota. **c** Representative microbiota with significant differences (*p* < 0.05), * Significant difference between antrum-predominant gastritis and *H. pylori* negative gastric antrum cancer patients. ^# ^Significant difference between *H. pylori* negative and positive gastric antrum cancer patients

**Fig. 5:**
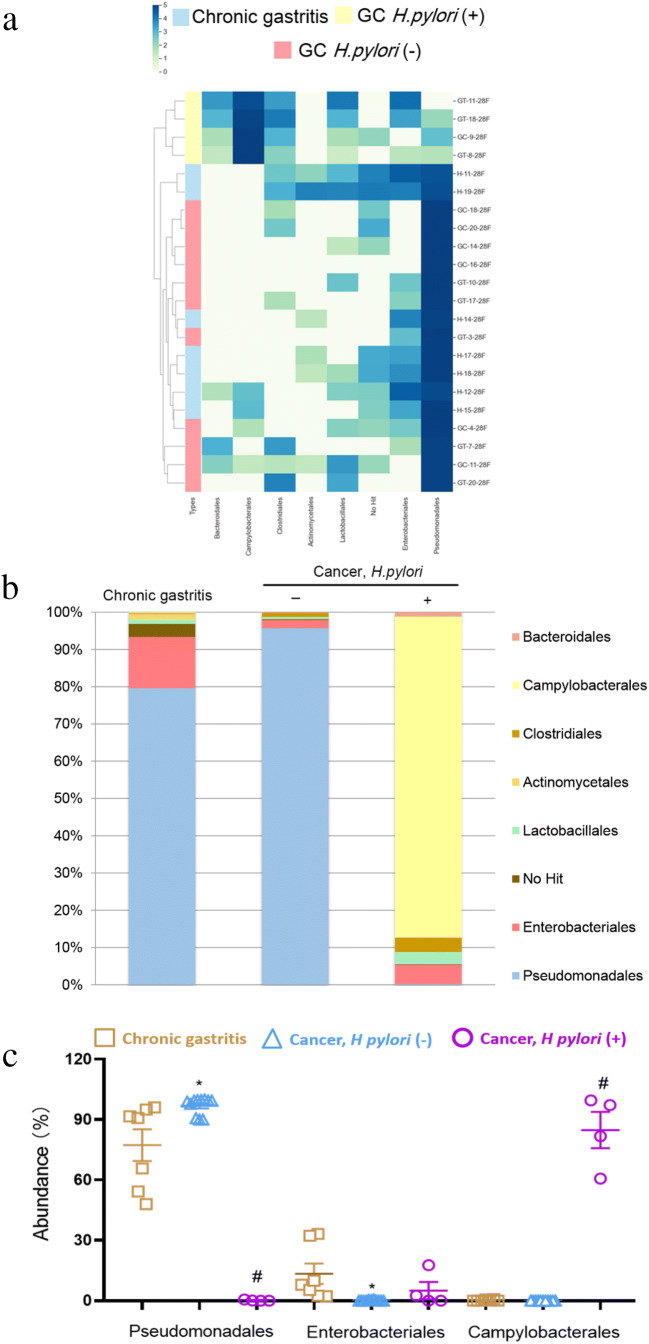
Microbial composition analysis of patients with corpus predominant gastritis and gastric corpus cancer. **a** Heatmap of gastric mucosa-associated microbiota with relative abundance over 1% in all subjects. **b** Bar plots of relative abundances of gastric mucosa-associated microbiota. **c** Representative microbiota with significant differences (*p* < 0.05), * Significant difference between corpus-predominant gastritis and *H. pylori* negative gastric corpus cancer patients. ^# ^Significant difference between *H. pylori* negative and positive gastric corpus cancer patients

Since we identified differences in the microbial composition in different anatomical sites in individuals with gastritis, we next examined how the microbiota profile in different anatomical sites may change from gastritis to gastric cancer. Overall, we found that there were only minor differences in major bacteria phyla between *H. pylori* negative gastritis and gastric cancer individuals from the corpus and antrum. Although the ranking of five main phyla differed slightly between these two anatomical sites, *Proteobacteria* was the dominant phylum in gastric cancer, with a slight increase compared to gastritis (Tables 2 and 3). At the order level, *Pseudomonadales* (96.87 ± 0.38% vs 77.34 ± 2.74%, *p* = 0.02) became the most abundant flora in gastric corpus cancer with a relative abundance > 90% in all subjects of this group (Fig. 5). The increase was more pronounced in gastric antrum cancer, *Pseudomonadales* was up to 50.48 ± 2.65% (vs 0.26 ± 0.36%, *p <* 0.01) with a relative abundance >50% in over half of all subjects (Fig.4). However, *Enterobacteriales* was less abundant in both gastric antrum (15.20 ± 1.70 vs 36.99 ± 3.65%, *p* = 0.01) (Fig. 4) and corpus (5.05 ± 1.83% vs 13.39 ± 1.79%, *p* < 0.01) cancer (Fig. 5). The proportion of *Actinobacteria* was found to be decreased in gastric antrum cancer in comparison to antrum predominant gastritis, while being over-represented in gastric corpus cancer when compared to gastritis in the corpus (Tables 2 and 3). A significant decrease in *Actinomycetales* was observed at the order level in gastric antrum cancer (15.59 ± 1.83% vs 0.07 ± 0.01%, *p* < 0.01) (Fig. 4), but not in corpus cancer.

**Table 2:** Relative abundances of major bacterial phyla in gastric antrum cancer

Taxonomy	Antrum gastritis (%)	GC *H. pylori* (−) (%)	GC *H. pylori* (+) (%)	*P* value
*Proteobacteria*	38.03	60.22	64.71	0.35
*Firmicutes*	36.94	33.11	23.04	0.76
*Actinobacteria*	14.63	0.22^a^	0.71	**0.01**
*Bacteroidetes*	2.95	0.93^a^	0.98	0.46
*Fusobacteria*	1.7	1.57	10.21	0.21

The *P* values are calculated using Mann-Whitney U test

^a ^Significant difference between *H. pylori* negative gastric antrum cancer patients and antrum-predominant gastritis

*P* value marked in bold if < 0.05

**Table 3:** Relative abundances of major bacterial phyla in gastric corpus cancer

Taxonomy	Corpus gastritis (%)	GC *H. pylori* (−) (%)	GC *H. pylori* (+) (%)	*P* value
*Proteobacteria*	80.18	82.8	91.22	0.85
*Firmicutes*	7.26	0.04	1.16	0.55
*Actinobacteria*	1.69	16.70^a^	7.47^b^	0.51
*Bacteroidetes*	1.36	0.01	0.11	0.52
*Fusobacteria*	0.38	0.01	0	0.15

The *P* values are calculated using Mann-Whitney U test

^a ^Significant difference between *H. pylori* negative gastric corpus cancer patients and corpus-predominant gastritis

^b ^Significant difference between *H. pylori* negative and positive gastric corpus cancer patients

Overall, we found that there are no gross differences in mucosa-associated microbiota profiles between chronic gastritis and *H. pylori* negative gastric cancer at the phylum level, while major changes occur at relatively lower taxonomic levels.

### Influence of *H. pylori* infection on microbiota profiles in gastric carcinoma

We next investigated variations in microbial community profiles to assess the potential impact of *H. pylori* infection on the presence of other bacterial flora in gastric cancer. The bacterial communities at the phylum level showed that the abundance of *Proteobacteria* was slightly higher under *H. pylori* infection status in both the antrum and corpus (Tables 2 and 3). Nonetheless, dramatic changes also occurred at the order level. Specifically, *Campylobacterales* (antrum: 45.00 ± 2.48%; corpus: 84.82 ± 3.88%), instead of *Pseudomonadales* (antrum: 2.65 ± 2.97%; corpus: 0.21 ± 0.26%), became the most abundant order in gastric cancer when *H. pylori* was positive (Figs. 4 and 5). These shifts were more noticeable in the corpus, in which all subjects showed a relative abundance of *Campylobacterales* higher than 90% (Fig. 5). The over-representation of *Actinobacteria* in gastric corpus cancer was decreased when infected with *H. pylori* (Table 3), but no additional significant changes were observed at the order level.

These results suggest that the influence of *H. pylori* on shaping the gastric cancer-associated microbiota profiles also occurs at lower taxonomic levels.

### Microbial community structures change from chronic gastritis to gastric carcinoma

**Fig. 6:**
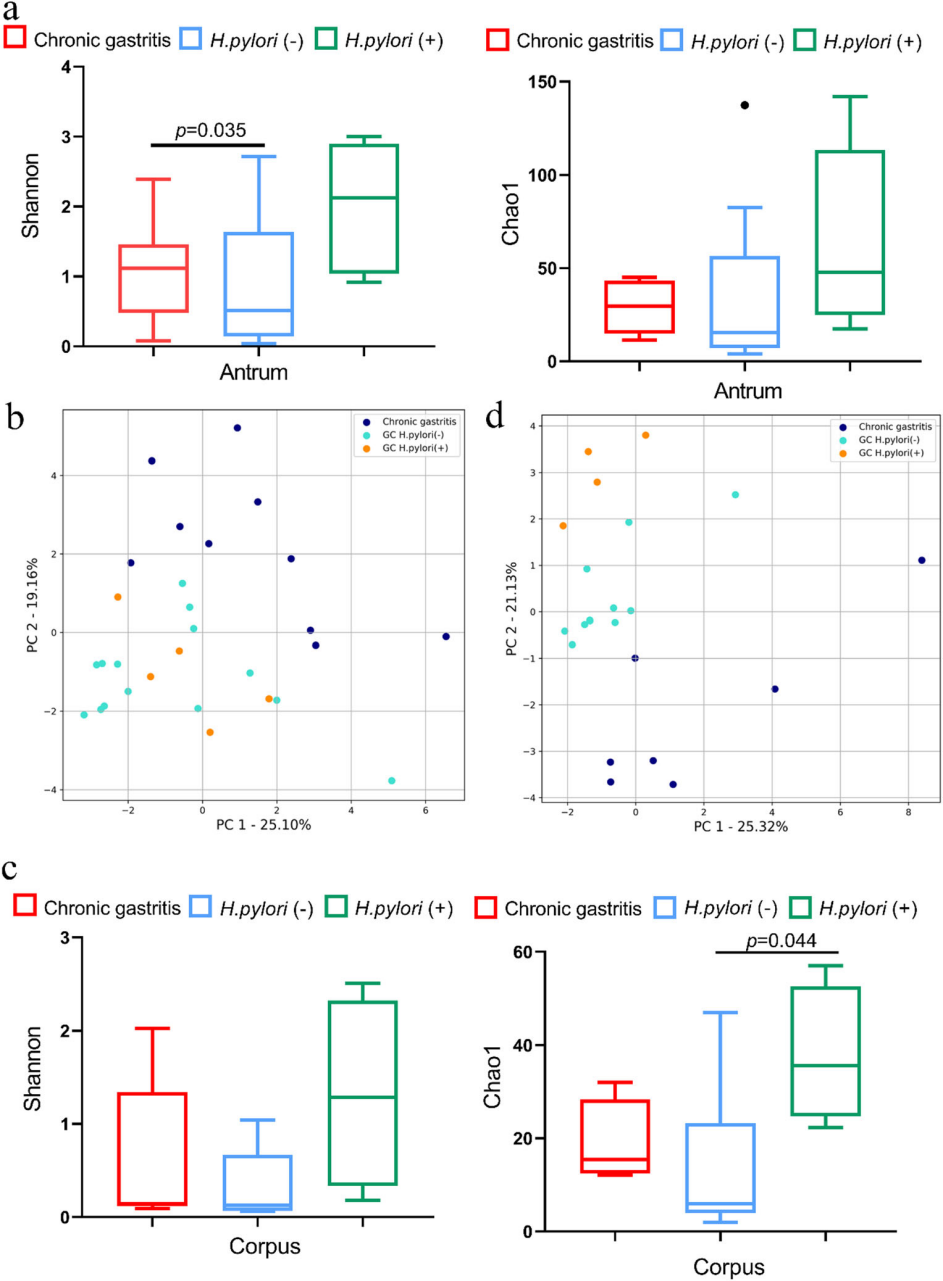
Microbial community structure analysis from chronic gastritis to gastric cancer. **a** Microbial alpha diversity and richness analyses between antrum predominant gastritis and gastric antrum cancer. **b** PCA analysis between antrum predominant gastritis and gastric antrum cancer. **c** Microbial alpha diversity and richness analyses between corpus predominant gastritis and gastric corpus cancer. **d** PCA analysis between corpus predominant gastritis and gastric corpus cancer

To evaluate alterations in the microbiota community structure from chronic gastritis to gastric carcinoma, we measured bacterial taxonomic richness, alpha diversity and beta diversity. We found that the Chao 1 estimated richness of microbiota in chronic gastritis was not significantly different from that in *H. pylroi* negative gastric cancer, irrespective of the anatomical sites (Fig. 6a, c). The alpha diversity Shannon index was markedly decreased in *H. pylori* negative gastric antrum cancer patients in comparison to antrum-predominant gastritis patients (Fig. 6a, *p* < 0.05), whereas only a downward tendency was observed in corpus mucosa (Fig. 6c). Next, the effect of *H. pylori* infection on microbial richness and alpha diversity was determined in gastric cancer. Microbial richness tended to increase under *H. pylori* infection status, but was statistically significant only in gastric corpus cancer (Fig. 6a, c). Alpha diversity tended to increase when infected with *H. pylori* both in gastric antrum and corpus cancer (Fig. 6a, c), but it did not reach statistical significance (*p* > 0.05).

Beta diversity analysis revealed an obvious separation between chronic gastritis and gastric cancer in both the antrum and corpus, but further distinguishing according to *H. pylori* infection status depended on the anatomical tumor sites (Fig. 6c, d). Gastric corpus cancer patients clustered separately based on the abundance of *H. pylori* (Fig. 6d), but not in patients with gastric antrum cancer (Fig. 6c).

These results suggest that there is no obvious alteration in microbial alpha diversity and richness in different stomach phenotypes, including chronic gastritis and *H. pylori* negative or positive gastric cancer. The endogenous bacterial community is disturbed during the initiation and development of gastric cancer, whilst the role of *H. pylori* in the progression of malignant tumors is limited, or at least needs to be further determined.

### Specific microbial taxa associated with gastric cancer

**Fig. 7:**
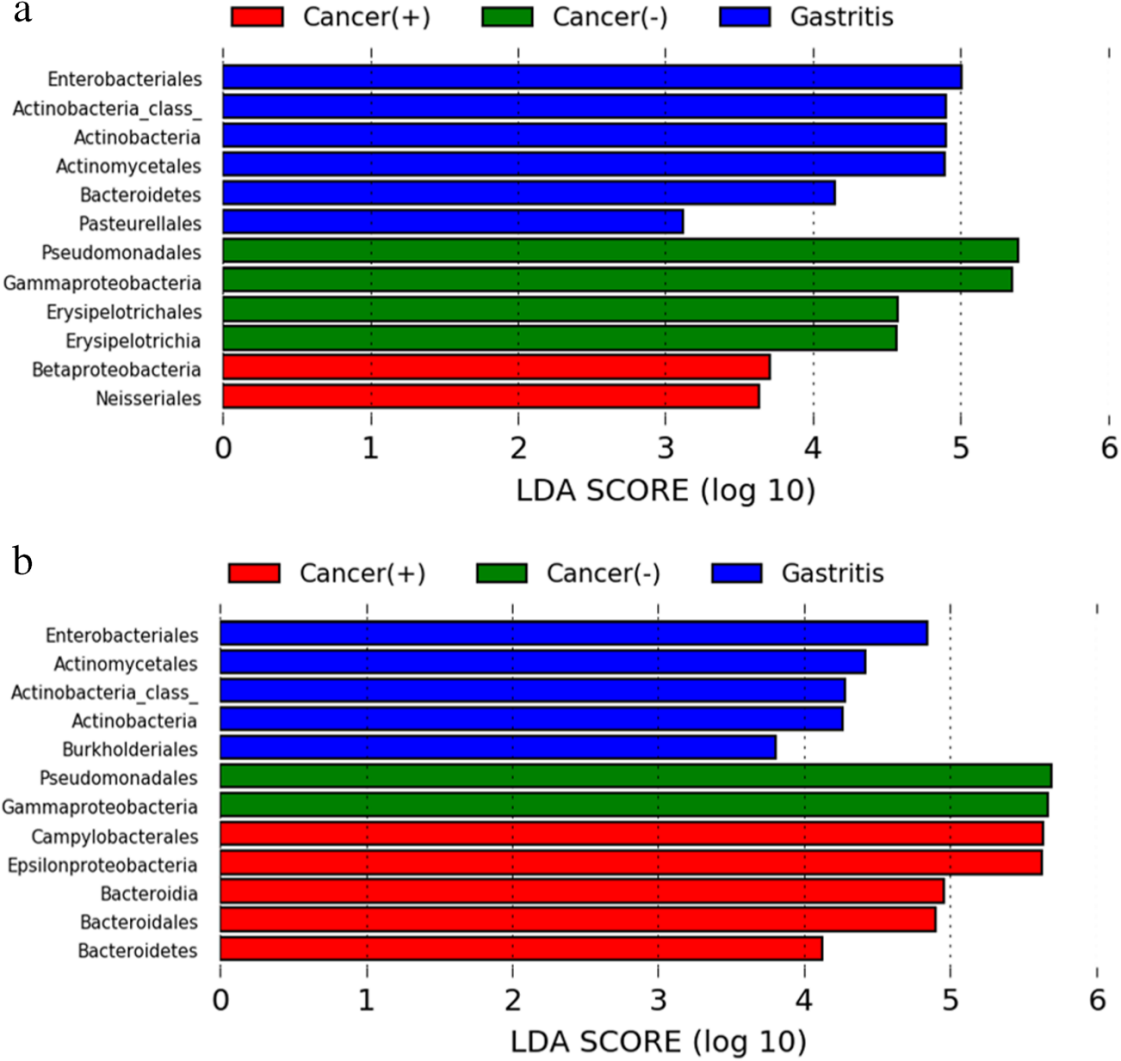
Influence of *H. pylori* infection on the specific taxa associated with different clinical diagnoses based on LEfSe analysis (LDA scores > 3 and *p* < 0.05). **a** Specific microbial taxa associated with antrum predominant gastritis and gastric antrum cancer. **b** Specific microbial taxa associated with corpus predominant gastritis and gastric corpus cancer

To identify the most relevant taxa responsible for each clinical diagnosis, we next performed LEfSe analysis at the order level and above, which has been validated for high-dimensional biomarker discovery [19]. For patients with antrum predominant gastritis and gastric antrum cancer, this analysis identified 12 taxa, including 5 orders, which were differentially abundant among the three groups. In *H. pylori* negative gastric cancer patients, an enrichment in *Proteobacteria* and *Firmicutes* taxa was observed, including the class *Gammaproteobacteria* with its specific order *Peudomonodales*, and the class Erysipelotrichia with its specific order *Erysipelotrichales*. However, in *H. pylori* positive gastric cancer patients, *Neisseriales* was the only identified enriched order within the class *Betaproteobacteria*. Additionally, *Actinomycetales*, *Enterobacteriales* and *Pasteurellales* were more abundant in the microbiota of patients with antrum predominant gastritis (Fig. 7a).

For patients with corpus predominant gastritis and gastric corpus cancer, there were also 12 specific taxa that were differentially abundant among the three different clinical diagnoses. In *H. pylori* negative gastric cancer patients, we only found that *Gammaproteobacteria* with its specific order *Peudomonodales* were significantly abundant. When it comes to *H. pylori* positive gastric cancer subjects, except *Proteobacteria* taxa with its specific order *Campylobacterales*, an enrichment in the phylum *Bacteroidetes* with its specific order *Bacteroidales* was observed (Fig. 7b). Moreover, *Enterobacteriales*, *Actinomycetales* and *Burkholderiales* were more abundant in patients with corpus predominant gastritis.

These analyses revealed the most relevant taxa responsible for differences between clinical diagnoses, and that the abundance of specific taxa depends on *H. pylori* infection status.

## Discussion

Through increasing technological developments, the microbial profiles in tumor environments are now being unraveled [20–23]. Here, we explored distinct gastric mucosa-associated microbiota profiles among different anatomical sites during gastritis, the shift of the microbiome community during gastric cancer progression, and the influence of *H. pylori* infection on the microbial community in gastric cancer. Our findings add to the understanding of associations between dysbiosis and gastric cancer development.

An intricate interaction between acid secretion and bacterial growth is a crucial determinant of *H. pylori* infection and gastritis progression [24–26]. The differential levels of acid secretion contribute to the distinct niches of the gastric antrum and corpus for microbial colonization [26]. In subjects with high acid production, *H. pylori* has been found to particularly colonize the gastric antrum and to lead to antrum-predominant gastritis. However, when acid secretion is impaired, the location of gastritis is dominant in the corpus [27]. Based on these observations, a detailed assessment of the microbial community at different anatomical locations is considered important in order to understand the role of the microbiota community in the stomach. Our results revealed similar gastric microbiota communities during chronic gastritis in three different anatomical sites. These findings are consistent with previously reported results indicating that the microbial diversity and richness of these three anatomical positions were similar [23] and that no significant correlation was observed between phylotype distribution and anatomical location in Chinese symptomatic upper gastrointestinal disease patients [28].

Changes in gastric acid secretion, local immune responses and nutrient availability contribute to the disruption of homeostasis, leading to disturbances in microbial composition, structure and function, resulting in the colonization and overgrowth of none-*H. pylori* bacteria [29]. The complexity of microbiota can be discerned based on biological similarity in order to analyze phylogenetic and evolutionary relationships between different phenotypes. Analysis at the phylum level may provide a general overview of distinguished characteristics of different diseases at a relatively higher taxonomic level. Our present study showed that the influence of *H. pylori* infection on gastric cancer mucosa-associated microbial composition at the phylum level was limited, and that microbiota in cancerous and non-cancerous tissues were both dominated by *Proteobacteria*, *Firmicutes*, *Actinobacteria*, *Bacteroidetes* and *Fusobacteria*, as reported before [21–23]. Significant differences among different groups mainly occurred at a relatively lower taxonomic level. Chronic gastritis was mainly characterized by enrichment of lactic acid producing bacteria such as *Lactobacillus* and commensals of the oral cavity and lower gastrointestinal tract including *Streptococcus*, *Citrobacter* and *Klebsiella* [30, 31]. These findings differ from previous studies, which reported that *Citrobacter* and *Klebsiella* were more abundant in gastric cancer and were associated with gastric carcinogenesis [22, 32, 33]. This discrepancy may be due to differences in ethnicity, sample type, dietary habits and/or environmental factors. When progressed into *H. pylori* negative gastric cancer, the microbial profiles shifted dramatically with an absolute abundance of *Pseudomonadales*, in which *Acinetobacter sp* was the only prevalent species. Consistent with our data, a previous report using public cancer genome sequencing data showed that *Pseudomonas* was the stomach adenocarcinoma-associated bacterium when excluding contamination attribution [34]. In contrast, when infected with *H. pylori*, *Campylobacterales* was dominating the mucosa-associated community in gastric cancer. At the species level, *H. pylori* was highly abundant within this order, especially in mucosa of the corpus. It is noteworthy that the nitrosating or nitrate-reducing bacterium *Neisseriales* was more abundant in gastric antrum cancer, which can promote the production of N-nitroso compounds (NOC) which, in turn, have been considered as potent carcinogens [32, 35, 36]. Enrichment of *Betaproteobacteria* and *Gammaproteobacteria* in gastric cancer indicated that none-*H. pylori Proteobacteria* may be closely associated with gastric carcinogenesis.

In our study, no significant alterations in local microbial community richness (evaluated by Chao 1) and alpha diversity (evaluated by Shannon index, except in the antrum) were observed during the development of gastritis to *H. pylori* negative gastric cancer. In addition, we found that the presence of *H. pylori* did not affect the species richness (except in the corpus) and alpha diversity of the gastric cancer community. This observation is not completely consistent with previous reports, showing significant reductions in microbial diversity and species richness in gastric cancer compared with chronic gastritis [22, 23]. Another cohort, in which *H. pylori* infection status was not considered, revealed that gastric cancer exhibited a significantly increased microbial diversity and species richness compared to non-cancerous tissues [21]. Factors that may provide an explanation to these discrepancies include variations in *H. pylori* status and virulence, sample type, dietary factors, as well as sequencing techniques and analysis methods used. Additionally, due to the different stages of gastric cancer development in our analysis cohort, a high degree of inter-subject variability may exist. Statistically significant differences among groups may, therefore, become apparent when the sample sizes are expanded. Nevertheless, beta diversity analysis revealed distinct microbiota structures between chronic gastritis and gastric cancer groups. The niche-specific microbial communities observed may reflect that multiple bacteria may have gradually adapted to disease-specific microenvironments. The microbial community structure could be diversified in gastric corpus cancer according to *H. pylori* infection status, but not in the antrum. This may be due to the different ambient pH levels between the corpus and antrum. As indicated above, the pH of the corpus may be more vulnerable to *H. pylori* infection than the pH of the antrum.

It should be noted that *H.pylpri* positive gastric corpus cancer showed a significant increase in species richness compared to the negative group. The alpha diversity also exhibited an increased tendency from *H. pylori* negative to positive gastric corpus cancer, although without statistical significance. This finding is supported by previous reports using paired cancerous and non-cancerous tissues, indicating that the *H. pylori* sequencing-positive group showed a significant increase in alpha diversity in comparison to the negative group [21]. Another study substantiated the view that *H. pylori* can markedly influence the diversity of gastric cancer [37]. Also, a mouse study indicated an increased bacterial diversity in *H. pylori *infected animals, which appears to correlate with observations made in infected humans [38]. A possible explanation for this phenomenon is that chronic infection of *H. pylori* induces an inflammatory cascade and gastric atrophy, a condition related to an impaired secretion of gastric acid leading to an increase in pH, consequently decreasing the load of *H. pylori* and its expression pattern, all together rendering a more tolerable environment for the colonization and outgrowth of non-*H. pylori* microbiota [39]. The suicide journey hypothesis of *H. pylori* through gastric carcinogenesis is supported by many reports, showing that the abundance of *H. pylori* is lower in any pre-malignant lesion or gastric cancer compared to that in non-malignant gastric mucosa [20, 22, 40]. Thus, *H. pylori* may be the predominant microorganism during the initial stages of gastric carcinogenesis, after which it becomes a minor component and substituted by new dominant microbiota, which favor gastric cancer development at later stages [41, 42]. What we observed in our study may lie in the earlier period of this shift. Understanding the timing of this evolution of gastric microbiota may provide insight into the intricate role of microbiota in tumor development. The best time at which to eradicate *H. pylori* and to administer a proton pump inhibitor should be determined in order to halt gastric carcinogenesis.

Our future studies will be aimed at analyzing *H. pylori* positive gastritis subjects and focusing on microbial changes from gastritis to gastric cancer with the presence of *H. pylori*, and fully clarify the role of *H. pylori* in gastric carcinogenesis. The current study was based on a relatively small number of patients, especially *H. pylori* positive gastric cancer patients. Future prospective follow-up studies using large populations are required to determine the landscape of gastric microbiota along with the development of gastric cancer. Nevertheless, our study provides a foundation for a more comprehensive understanding of gastric cancer mucosa-associated microbiota and the complex interplay between *H. pylori* and other microbiota, which may shed light on the pathogenesis, diagnosis, treatment and prevention of gastric cancer.

## Electronic supplementary material

Fig. S1 Microbial compositional analysis at the species level. Representative microbiota with significant differences (*p*<0.05) from patients with a chronic gastritis of three different anatomical sites b antrum predominant gastritis and gastric antrum cancer c corpus predominant gastritis and gastric corpus cancer (PNG 470 kb)

High resolution image (TIF 12.6 mb).

## Data Availability

The data and code used to support the findings of this study are available from the corresponding author upon request.
