# Supplementary figures and images for: Alterations in mucosa-associated microbiota in the stomach of patients with gastric cancer
Source: Cell Oncol (Dordr). 2021 Mar 26;44(3):701–14. doi: 10.1007/s13402-021-00596-y (PMC8213677; doi:10.1007/s13402-021-00596-y)

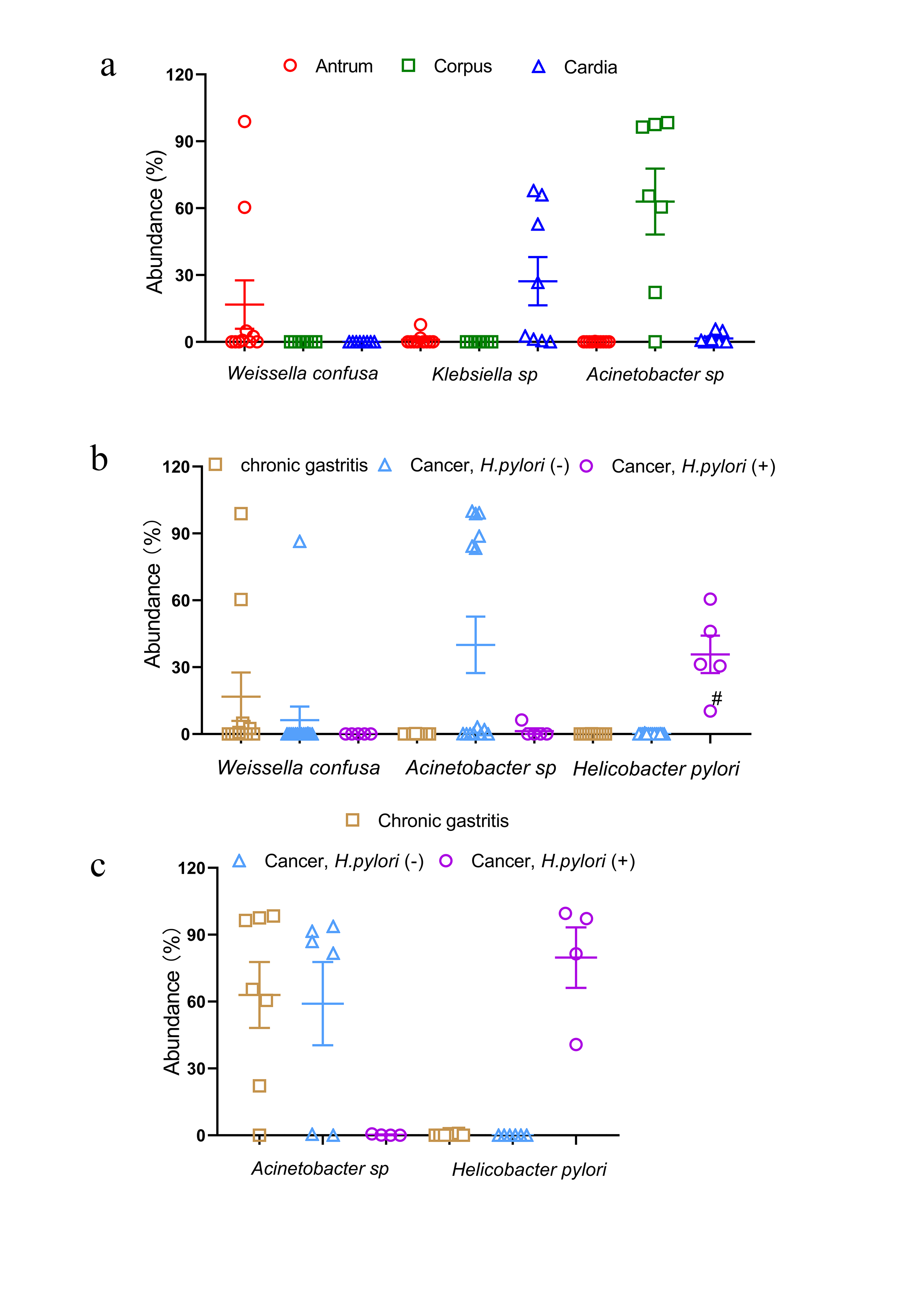

Supplement: Supplementary file 1 — Microbial compositional analysis at the species level. Representative microbiota with significant differences (p<0.05) from patients with a chronic gastritis of three different anatomical sites b antrum predominant gastritis and gastric antrum cancer c corpus predominant gastritis and gastric corpus cancer (PNG 470 kb) [file 13402_2021_596_Fig8_ESM.png]

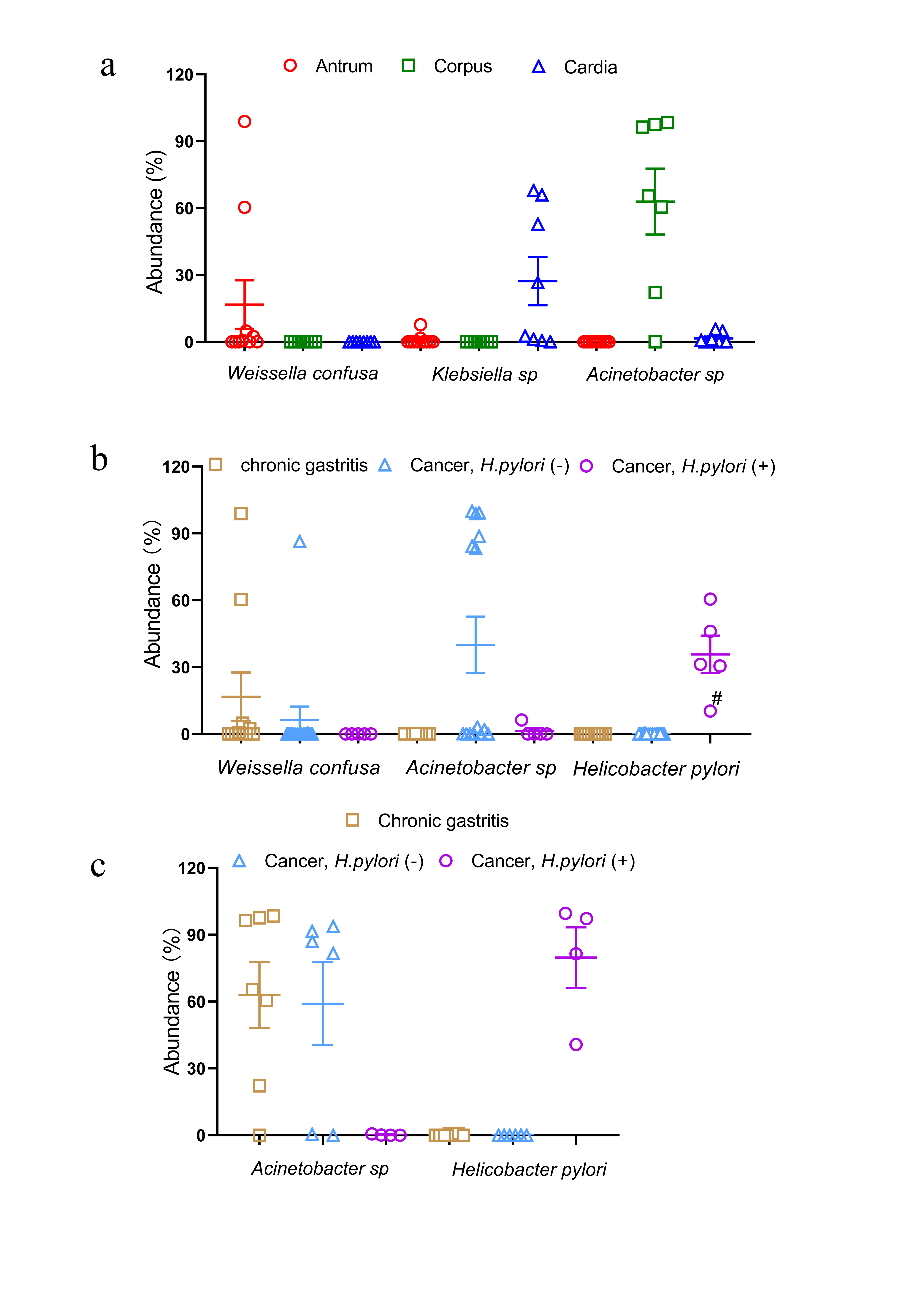

Supplement: Supplementary file 2 — High resolution image (TIF 12.6 mb). [file 13402_2021_596_MOESM1_ESM.tif]
